# Systematic online living evidence summaries: emerging tools to accelerate evidence synthesis

**DOI:** 10.1042/CS20220494

**Published:** 2023-05-23

**Authors:** Kaitlyn Hair, Emma Wilson, Charis Wong, Anthony Tsang, Malcolm Macleod, Alexandra Bannach-Brown

**Affiliations:** 1 Centre for Clinical Brain Sciences, The University of Edinburgh, Edinburgh, U.K.; 2 Anne Rowling Regenerative Neurology Clinic, University of Edinburgh, Edinburgh, U.K.; 3 Euan Macdonald Centre for Motor Neuron Disease Research, University of Edinburgh, Edinburgh, U.K.; 4 King’s Technology Evaluation Centre, King’s College London, U.K.; 5 Charité Universitaetsmedizin Berlin, Berlin Institute of Health – QUEST Center, Berlin, Germany

**Keywords:** automation, data mining, evidence-based medicine, machine learning, systematic review

## Abstract

Systematic reviews and meta-analysis are the cornerstones of evidence-based decision making and priority setting. However, traditional systematic reviews are time and labour intensive, limiting their feasibility to comprehensively evaluate the latest evidence in research-intensive areas. Recent developments in automation, machine learning and systematic review technologies have enabled efficiency gains. Building upon these advances, we developed **S**ystematic **O**nline **L**iving **E**vidence **S**ummaries (SOLES) to accelerate evidence synthesis. In this approach, we integrate automated processes to continuously gather, synthesise and summarise all existing evidence from a research domain, and report the resulting current curated content as interrogatable databases via interactive web applications. SOLES can benefit various stakeholders by (i) providing a systematic overview of current evidence to identify knowledge gaps, (ii) providing an accelerated starting point for a more detailed systematic review, and (iii) facilitating collaboration and coordination in evidence synthesis.

## The need for systematic review in biomedicine

For hundreds of years, researchers have recognised the need to effectively synthesise research evidence to facilitate evidence-based decision making [1]. Systematic review (SR), a research methodology to locate, select, evaluate and synthesise all evidence on a specific research question based on pre-specified eligibility criteria, emerged in the latter half of the twentieth century as a tool to enable clinicians to make evidence-based decisions about patient care. Evidence from SRs is widely regarded as the highest level of evidence in the evidence hierarchy and is often used to inform improvements in clinical trial design and health care [2]. Over the past decade, there have been growing attempts to apply similar methodologies to summarise and evaluate findings from animal (*in vivo*) and, more recently, *in vitro* laboratory research [3]. Many challenges – including the internal and external validity of preclinical research – may impede the translation of findings from animal and cell-based models to human patients. Preclinical SRs can help expose the reasons behind this discrepancy [4]. For example, poor methodological quality and publication bias in stroke research may overestimate therapeutic benefits observed in animal models [5,6]. SRs of including both clinical and preclinical literature can be helpful to inform selection and prioritisation of candidate drugs in clinical trials, especially in fields with high rates of translational failure such as neurodegenerative diseases [7–9].

## Barriers limiting the use of systematic reviews

There are proven benefits of conducting SRs for evidence-based research and decision making. Here, we focus on the application of SRs to preclinical and clinical evidence. However, traditional SR methodology is no longer optimal for the current research landscape where rates of publication are increasing tremendously [10]. Here, we outline the three key barriers to impact.

### Failing to capture all relevant evidence

One of the key strengths of SRs over more traditional narrative literature review is that they aim to capture all the available evidence. However, even SRs are not universally successful in this regard [11–13]. Methodological choices such as inadequate search strategies, narrow research questions and exclusion of grey literature sources (e.g., preprint servers), and factors relating to lack of standardisation in database indexing and delays in indexing, all limit the completeness with which relevant primary research is identified. Systematic searches typically search for specific terms within keywords, title and abstract within bibliographic databases. However, emerging data from ongoing SRs of open field test behaviour and synaptic plasticity in transgenic Alzheimer’s models which used full-text searching found that 82% and 69% of relevant manuscripts, respectively, were not identified by searching the keywords, title or abstract [14]. Similarly, in a SR of oxygen glucose deprivation in PC12 cells, human reviewers conducting title and abstract screening erroneously excluded 14% of relevant studies, which were only identified using a broader search coupled with full-text screening [15]. Traditional approaches therefore may not be sensitive enough for preclinical biomedical SRs.

### Failing to keep up with the literature

By the time of their publication, SRs are frequently many months or years out of date [16,17]. Manually searching bibliographic databases, screening studies for relevance, and extracting study details is laborious and time-consuming [18]. This issue is amplified in highly research intensive areas, where there could be thousands of potentially relevant studies published every year. For example, to update a review of animal models of neuropathic pain, we performed a systematic search three years after the original and found 11,000 new publications to screen for relevance [19]. Furthermore, updating SRs to incorporate recent evidence is not standard practice [20] and can require just as much effort as conducting the initial review [21,22]. Incorporating new studies into evidence summaries is an ongoing challenge with the rate of publication increasing exponentially each year [8]. Even where updates to SRs are done, they may use different SR methodology [23] making it difficult to contextualise the new findings. For research areas with a high rate of publication, it has been suggested that the median ‘survival’ of reviews (i.e., period over which they remain up to date) is just under three years [24]. Recent findings suggest that half of Cochrane’s reviews were published more than 2 years after the original protocol, and that this gap may be widening over time [25]. It has also recently been reported that 85% of Cochrane’s reviews have not been updated for over 5 years [26].

### Failing to collaborate and co-ordinate efforts

Without co-ordinated efforts to prioritise and conduct which SRs should be done, the degree of overlap can be significant [27], with little acknowledgement of findings from previous reviews on the same or similar research questions [28]. Collaboration among researchers can help reduce the resource burden required to complete a SR, but this requires researchers to be aware of other reviews being conducted in areas that overlap with their own interests. Greater use of registration tools such as PROSPERO is likely to reduce duplication of effort. More cohesive evidence synthesis communities, with stronger collaboration between primary researchers, information professionals, statisticians and evidence synthesists and key stakeholders and users of evidence synthesis research, would help to address this. Stronger communities could improve how priority questions in biomedicines are identified and addressed, ensure that synthesis research is conducted collaboratively, and increase the likelihood that findings are put into practice in a timely manner [29].

## Emerging approaches and ‘living’ systematic reviews

Setting aside the usefulness of SRs and meta-analyses, alternative approaches such as rapid reviews, or scoping reviews [30] may be sufficient and perhaps more feasible in a given timescale. Evidence gap maps are an emerging concept, where all evidence relating to a broad research question is collected and presented in a user-friendly format, to identify knowledge gaps and inform future research priorities [31,32].

Recently, several groups have sought to facilitate faster evidence synthesis through the application of automation technologies [33]. By incorporating natural language processing and other machine-assisted techniques, the human effort and time required to complete each step of the SR process can be dramatically reduced. Several tools have been developed for performing systematic searches [34,35], record screening [36–38], deduplication [39] and extracting information from publications [40,41]. Through levering such methodologies, the concept of ‘living’ SRs has been proposed as now being a feasible approach [42]. A living SR is a systematically obtained and continually updated summary of a research field, incorporating new findings as and when they emerge. However, due to the heterogeneity of published literature, some human effort will likely always be required to understand and extract detailed information and numerical data from publications.

## Systematic Online Living Evidence Summaries (SOLES)

Drawing on the objectives of living SRs and evidence gap maps, and considering the barriers faced by conventional SR approaches, we introduce a new approach for evidence synthesis. **S**ystematic **O**nline **L**iving **E**vidence **S**ummaries (SOLES). SOLES are systematically obtained, continuously updated, curated, and interrogatable datasets of existing evidence, enabled by automation. They are likely of greatest benefit to research-intensive areas with high rates of publication, as seen in much of biomedicine. Here, we describe where SOLES fit into the evidence synthesis ecosystem (see Table 1), describe an exemplar SOLES approach as applied to Alzheimer’s disease research (see Table 2), discuss how SOLES may address some of the barriers limiting traditional SRs, and discuss current limitations and future developments of the SOLES approach.

The major differences between SOLES and related approaches lie in the scope of evidence included and the application of automation technologies. Most living SRs have a narrow, clearly defined research question, whereas SOLES aim to summarise much larger areas of research. SOLES and evidence gap maps share many similarities in their overall approach and immediate use-cases, however SOLES encompass broader research domains and aim to present a heterogeneous range of visual summaries, trends, and functionality to research users. A comparison of each approach is shown in Table 1. Importantly, evidence gap maps have largely been applied to collate evidence from clinical studies from the social sciences, where SRs are routinely conducted. As such, evidence from existing SRs are an important feature. SOLES were developed with the initial aim of curating the vast quantity of diverse evidence in preclinical models of human disease to facilitate SRs in this area and drive research improvement.

**Table 1:** Summary of the features and differences between SOLES, evidence gap maps and living SRs

	SOLES	Evidence gap maps	Living systematic review
Systematic search	Broad domain-specific search terms	Search terms tailored to a broad research area or question.	Narrower search terms, tailored to a specific research question
Eligibility criteria	Broad inclusion criteria including all experiments of relevance to research domain	Inclusion criteria focusing on a particular research question or area, typically include SRs on the topic	Narrower inclusion criteria, focusing on a particular population, intervention and/or outcome
Data annotation	Prioritised annotation of major PICO elements, study quality indicators and other measures (including transparency of reporting)	Prioritised annotation of major PICO elements and study quality indicators	In-depth annotation of all PICO elements, study quality indicators, study design characteristics and other metadata
Meta-analysis	Not currently a feature	In most cases, not appropriate	May be conducted in the context of systematic review if appropriate
Output	An online dashboard with several interactive visual summaries of the latest evidence	A visual overview of evidence (i.e., interventions and outcomes) relevant to research question or area	A detailed synthesis and evaluation of evidence related to a pre-defined, narrow research topic often in the form of a scientific publication
Use cases	Used to gain a high-level overview of a research domain, to identify heavily studied areas or gaps in knowledge, to benchmark quality and transparency measures and to serve as a starting point for more detailed SRs on narrower topics	Used to gain a high-level overview of a research question, to identify heavily studied areas or gaps in knowledge	Used to make decisions regarding future research (evidence-based research), and to inform policy and practice
Updates	Updated continuously in response to emerging evidence (e.g., daily or weekly)	Updated frequently (as described in an individual protocol)	Updated frequently (as described in an individual living systematic review protocol)

Abbreviation: PICO: Population, Intervention, Comparator and Outcome.

**Table 2:** Exemplar SOLES project – AD-SOLES workflow

Stage	Details	Integrated tools
Systematic search	● Searches conducted using general Alzheimer’s disease search terms across Web of Science, Scopus, EMBASE and PubMed ● Customised R packages which make use of APIs to retrieve data on a weekly basis for three databases, while EMBASE is searched manually every 6 months ● Additional metadata for each study retrieved from CrossRef and OpenAlex	rcrossref [46] openalexR [47] RISmed [48] ScopusAPI [49] rwos [50]
Automated deduplication	● When new citations are retrieved, a check is performed to see if they already exist in the SOLES database ● Automated deduplication tool identifies duplicates between new citations and existing citations and within new citations (across databases)	ASySD [39]
Machine-assisted screening	● Trained machine classifier (hosted at EPPI Centre, University College London) determines likelihood of a citation describing experiments in AD animal models	Machine classifiers [38]
Automated data annotation	● Full texts downloaded using open access APIs and publisher (dependent on institutional access) APIs ● Risk of bias reporting assessed using automated tool ● Open access status determined using CrossRef and OpenAlex APIs ● Open data and code status determined by X tool	rcrossref [46] pre-rob tool [41] openalexR [47] ODDPub [51]
Interactive shiny app	● Shiny application available at https://camarades.shinyapps.io/AD-SOLES/ ● This allows users to:	
	1. Gain an overview of the number of relevant publications in different categories, and intersections of those categories	
	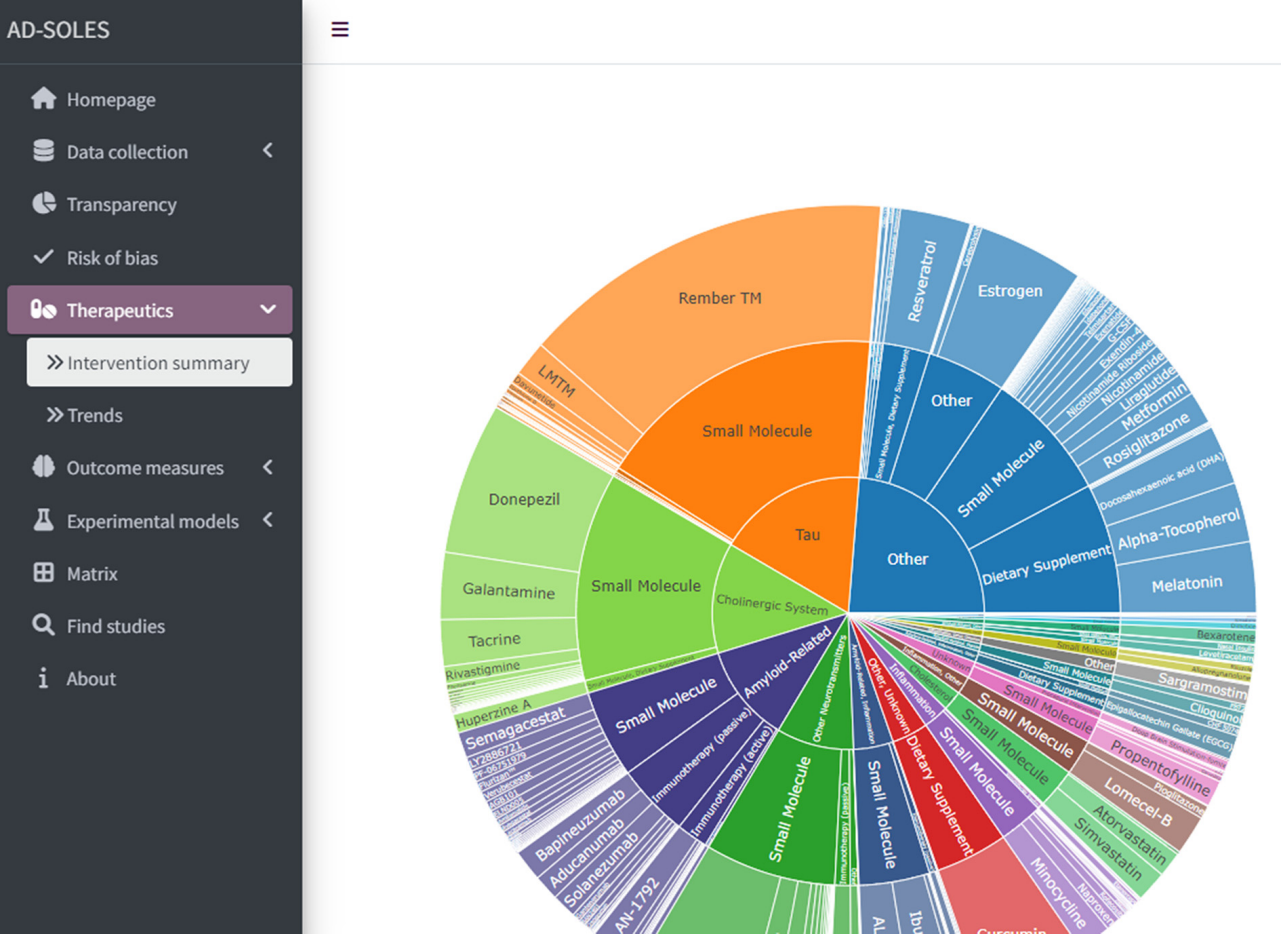	
	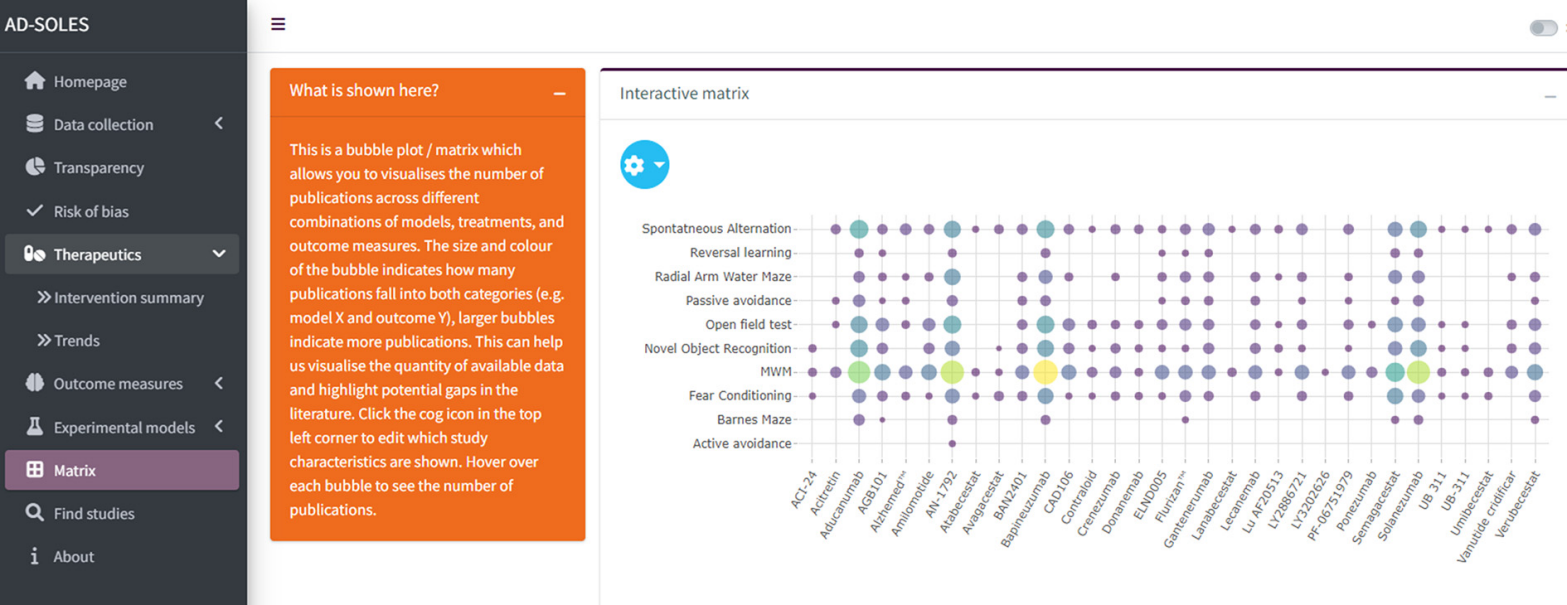	
	2. Visualise the proportion of publications with certain transparency and quality measures over time	
	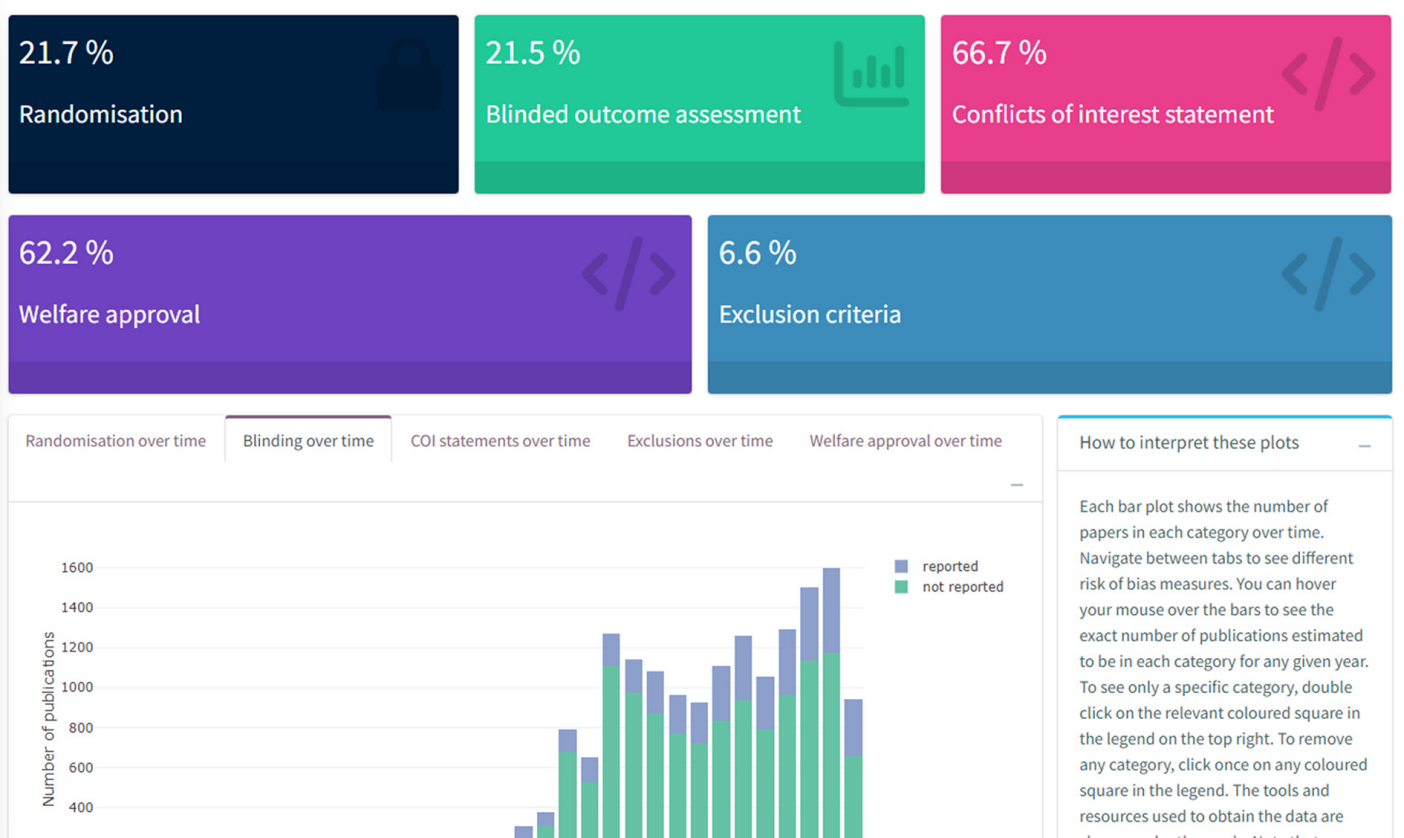	
	3. Download a highly filtered citation lists to start a more detailed systematic literature reviews	
	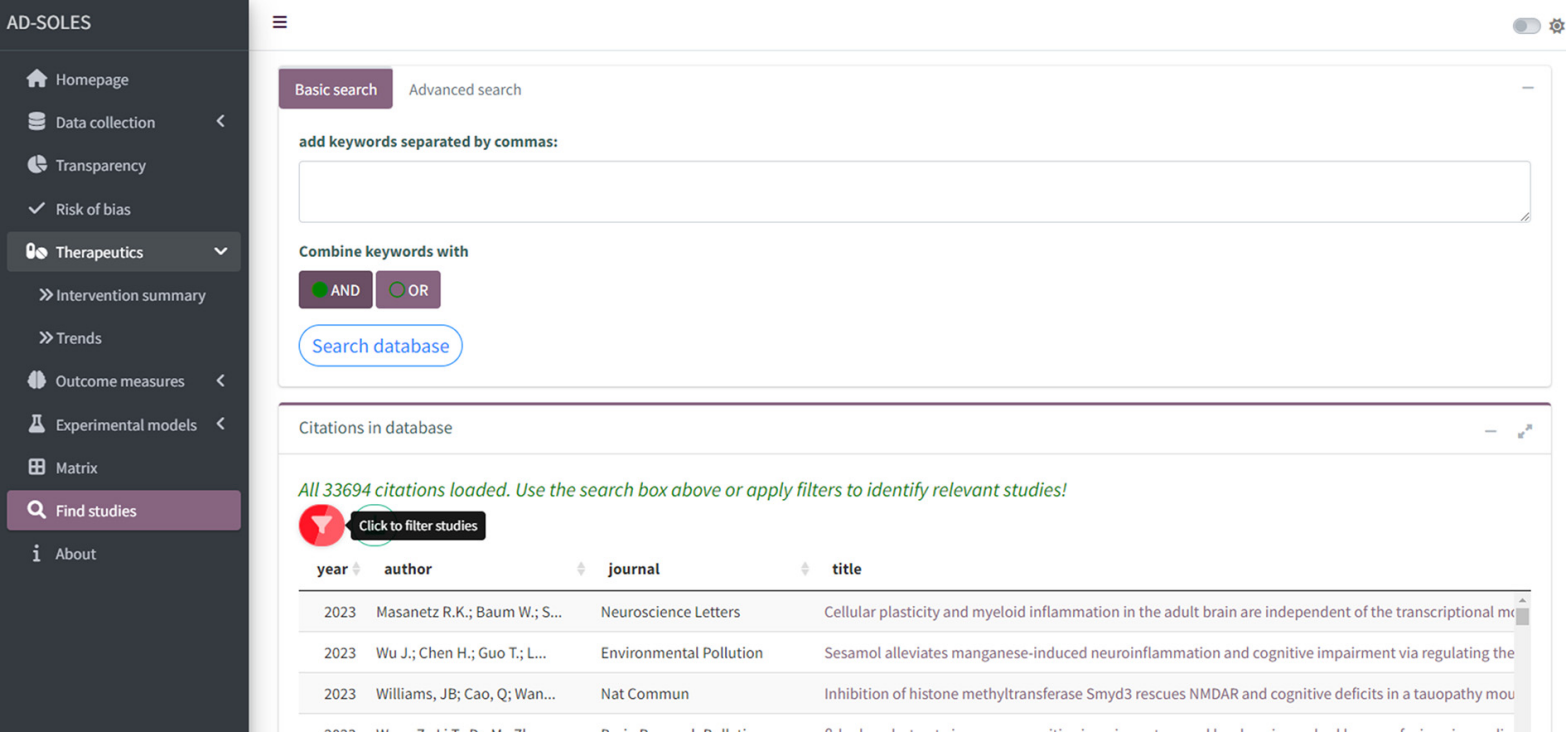	

**Figure 1:**
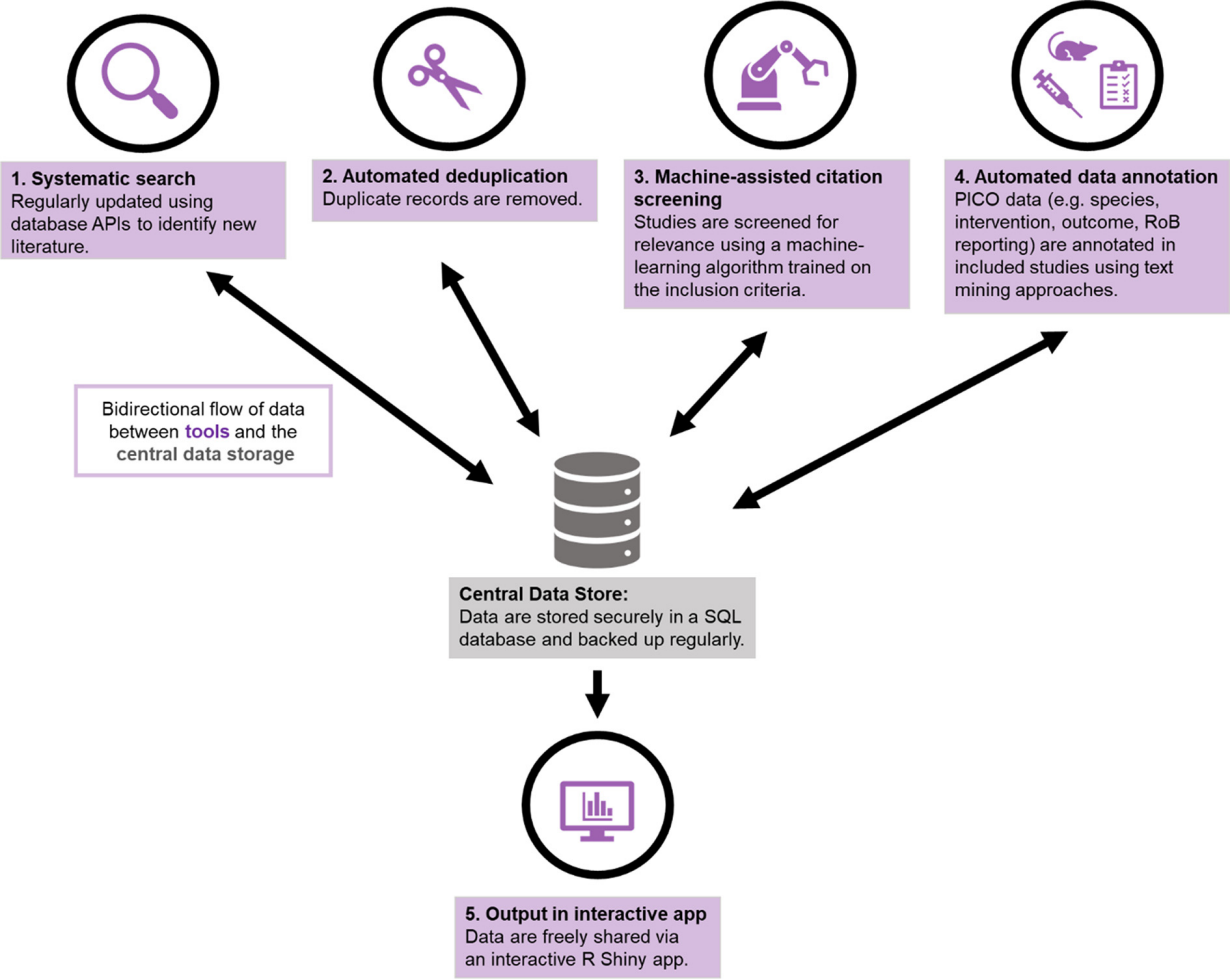
The automated SOLES workflow

The basic workflow for a SOLES is illustrated in Figure 1. SOLES begin with a wide search strategy across several biomedical databases and use less stringent criteria for inclusion – for example, including all laboratory experiments in any animal model of Alzheimer’s disease. Full texts of included studies are retrieved – for open access publications via Unpaywall, and for others via institutional subscriptions using Crossref [46–50]. High-level data are then extracted to categorise the research – e.g., by disease model(s), intervention(s), and outcome measure(s). Much of this process can be automated, either through machine learning approaches or simple text mining e.g. [51]. In addition, high-level quality measures may also be extracted, with quality of reporting assessed using text-mining or machine learning tools (e.g., [41,43]). This curated dataset and accompanying visualisations can then be made accessible to the research community via an interactive web application, such as R Shiny Apps (RRID:SCR_001626). This enables key stakeholders such as researchers, regulatory agencies, and funders to quickly ascertain the quantity and quality of research evidence, both overall and within each subgroup of studies employing each model, testing a certain treatment, or measuring a specific outcome. Furthermore, if the SOLES dataset sufficiently captures the existing literature, those wishing to perform a SR within that research domain can download a filtered set of references as a starting point, eliminating one of the most time-consuming tasks in the systematic review process [12].

The SOLES approach integrates automated processes to benefit end users. Despite the existence of automation tools, as summarised earlier in this paper, there are significant practical and knowledge barriers which reduce their uptake by individual SR teams [44]. Many tools exist as isolated use-cases which lack integration with other parts of the systematic workflow and may not be immediately available to researchers without sufficient technical expertise [45]. SOLES provides a single platform that integrates several tools in a pipeline to benefit researchers, evidence synthesists, and evidence users and decision makers (e.g., funders and regulatory agencies). There are direct benefits in obtaining a single evidence base on a topic, through a concentrated and collaborated effort, on which further, more detailed, reviews and primary research may be based.

## Maximising the potential of research evidence

SOLES also aim to transform the way we make sense of, and evaluate, research evidence. Next, we discuss how the SOLES approach can offer solutions to some of the barriers which limit more traditional SRs and benefit key stakeholders of evidence.

### Capturing a wider range of evidence

Developing search strategies for SRs has always been a balancing act between trying to ensure that no relevant evidence is missed (i.e., the search has high sensitivity) without capturing too many irrelevant publications to screen (i.e., the search has high specificity). Using automation approaches for record screening allows for broader systematic search strategies with higher sensitivity, to prioritise sensitivity over specificity. At the most extreme, one might imagine record screening being applied to an entire bibliographic database rather than to the results of a systematic search, the specificity being derived from downstream, automated tasks applied to full text articles. However, current computational limitations and tool performance mean that this is not possible at present.

Further, the sensitivity of the SOLES approach to literature searching does not rely on the presence of specific PICO elements in the title or abstract of publications. The pool of potentially relevant publications is much larger but, as we have shown in exemplar SOLES projects, the evidence can then be further subdivided into PICO categories by applying natural language processing techniques (regular expressions or machine learning algorithms) on the full-text of the publication.

A highly sensitive search to retrieve as many relevant citations as possible comes with the caveat that many individual citations will have been identified from several databases. With extremely large searches, this issue is amplified and difficult to manage using conventional deduplication tools. We have recently developed ASySD – the **A**utomated **Sy**stematic **S**earch **D**eduplication tool [39]. ASySD is an open-source tool created with SOLES functionality in mind, and allows the user to preferentially retain certain citations within a group of duplicate citations. For example, users can elect to retain older citations already present (and potentially annotated) in the dataset, or to retain the record with the most complete information.

Using SOLES as a starting point for obtaining the latest and most relevant research enables researchers and funding agencies to quickly gain a credible and systematic overview of current evidence. This can facilitate decision making, ensuring that future research is focused on the areas of greatest need and minimising research waste.

### Keeping up to date with the literature

A major goal of SOLES projects is to synthesise new research evidence as soon as it becomes available, thus preventing any delay in making use of that evidence to inform future research and decision making. SOLES developers can implement fully automated searches for publications using accessible Application Programming Interfaces (APIs) – which *exist* for several platforms including PubMed (NBCI), Scopus, Dimensions and Web of Science collections – and schedule these search queries to run at scheduled time periods. Attempts to automate some searches have been hampered by issues in retrieving citation data of the same quality (with abstracts and other meta-data present) and cost for access. Depending on institutional access and specific database needs, this step may require some human input. The retrieval of new citations on a regular basis also facilitates attempts to update SRs with minimal additional effort.

Systematic review questions generally focus on specific PICO (population, intervention, comparison and outcomes). To enable SOLES users to identify the latest relevant research, categorisations can be added to publications using regular expressions (regex) search dictionaries for animal models, interventions and outcome measures. A regex is a sequence of characters relating to a pattern of text, and are commonly used in library and information science and more widely. Overall, these approaches are time-saving and can be highly sensitive, especially if sufficient effort is placed into designing regex probes to account for common synonyms and deviations in punctuation and spelling (Bahor et al., 2017). In this way, using a curated citation data set derived from a SOLES platform can be an accelerated starting point for a more detailed SR.

To ascertain study quality, and to gain insights over whether primary study quality may be improving over time, similar text-mining tools can be applied to research articles to measure reporting quality or measures to reduce the risk of bias [43]. More recently, machine learning classifiers tailored to preclinical research have been developed to assess risk of bias reporting [41].

The automated PICO annotation and quality assessment tools allow for flexibility of the approach to analysis, depending on the needs of the research user. For example, the quality of a whole domain (e.g., animal research in Alzheimer’s disease) of evidence can be quickly determined for funders making decisions in the design of new funding calls. Alternatively, current data on highly specific research questions can be interrogated by primary researchers designing a new experiment. Together, these approaches bring us a few steps closer to making use of new evidence at a much quicker pace. Further, measuring research quality indicators in an automated way may allow for the benchmarking of research improvement activities over time [52,53].

### Collaboration and co-ordination

SOLES approaches require substantial human effort to set-up and ensure that the automated processes used to screen and annotate information function optimally. Many of the automation tools used to power SOLES require initial human input. For example, record screening uses supervised machine learning algorithms, requiring in turn large, curated training datasets. A random subset of search results should be screened manually to train an algorithm on the inclusion and exclusion criteria of a particular SOLES project, even if such an algorithm has already been validated in a different context. This calls for efficient collaboration and co-ordination to ensure that training data can be collected quickly, and that data are reliable enough to train the algorithms.

Large projects and collaboration provides an opportunity to use a crowdsourcing approach, which, if well-co-ordinated, allows for quicker data collection. Crowdsourced approaches work best if participants are given ‘micro-tasks’ to perform, such as screening or annotation of only a few pieces of information at a time [54]. Participating in a crowdsourced project can be beneficial, especially for junior researchers or students who want to get involved in systematic review but do not have the time or skills to conduct a full review themselves. However, it is important to consider whether you have enough resources to train, manage and enthuse your crowdsourced participants during a project.

Further collaboration can be used to establish the creation of new SOLES projects, ensuring that domains of most interest to researchers and funders are prioritised, and maximising crowd engagement to generate ideas for model, intervention and outcome assessment ontologies.

Furthermore, the ability of SOLES to provide a continually updated, comprehensive, annotated summary of current evidence, uncertainties and gaps of an entire research domain may prove instrumental in evidence-based policy making and research priority setting where broad-scope evidence synthesis produced within the timeframe of policy cycles are preferrable over traditional SRs which typically emphasises accuracy and depth of a narrower field [55].

## Evolution of SOLES

SOLES projects are an emerging methodology, and we have thus far focussed our efforts on synthesising evidence from preclinical laboratory experiments within research-intensive areas where there is an urgent need to develop new therapeutics.

The evolution of the SOLES approaches began with a SR of animal models of depression which used machine learning for title and abstract eligibility screening and text-mining techniques to automatically extract key methodological details from publications including the type of animal model used and the pharmacological interventions tested [56]. The final categorised dataset of over 18,000 publications was then displayed in an interactive web application for others to interrogate.

Later, we expanded the SOLES approach to integrate more automation technologies, such as study quality and risk of bias annotation using a regular expressions approach, in an early version of our Alzheimer’s disease dashboard. Further, by implementing a crowd-sourced approach to build up sets of annotated data at a quicker pace, we could train tailored automation approaches. Using this approach, we created a pilot COVID-SOLES, a resource which was updated on a weekly basis with new evidence in the emerging pandemic [57]. More recently we created the SPRINT-SOLES, in collaboration with over 20 experts in plant protection products who annotated subsets of the literature to use as training data for automated tools [58]. Harnessing this resource allowed researchers to produce rapid reviews of the evidence on the harms of pesticide exposures across clinical epidemiology, ecosystems and laboratory research. Our latest SOLES project, AD-SOLES [59], is the first example of a fully automated workflow from citation collection, screening for relevance, and organising data by risk of bias reporting, transparency measures, and study design characteristics in the field of Alzheimer’s disease research.

In parallel, we have created MND-SOLES-CT [7,60,61] to inform selection and prioritisation of candidate drugs for evaluation in clinical trials in motor neuron disease (MND). MND-SOLES-CT reports current curated evidence from Repurposing Living Systematic Review – MND, a three-part machine learning assisted living systematic review of clinical studies of MND and other neurodegenerative diseases which may share similar pathways, MND animal *in vivo* studies and MND *in vitro* studies.

Bringing together the advances and learning points from each of these projects, we are now in the position to develop a functional automated workflow that can be replicated to form the skeleton of all new and ongoing SOLES projects. Our early SOLES approaches made great use of the R programming language and R Shiny Apps (RRID:SCR_001626). As the technological functionality has evolved, subsequent SOLES approaches have drawn in more programming tools such as database APIs, SQL databases and tools built in Python (RRID:SCR_008394), capitalising on interoperability.

## Limitations of the SOLES approach

There are several limitations within the automated SOLES approaches we have outlined. Publications with no indexed abstract text may be incorrectly handled by machine learning-assisted screening approaches, while publications with no accessible full-text documents cannot be further categorised programmatically. The evolution of scientific publishing towards open-access publishing and improvements to the way citations are indexed in biomedical databases (e.g., to include abstracts with every publication in bibliographic databases) will support evidence synthesis approaches such as SOLES moving forward. The limitations of supervised machine learning algorithms for record screening of large corpuses have been discussed elsewhere (Bannach-Brown et al., 2019).

A further barrier to a fully automated approach, is that the way preclinical research publications are currently structured does not allow for accurate detection of PICO elements for every experiment. A publication may describe several experiments in different animal models, testing different treatments and measuring those animals on different outcomes. Further, it is difficult to determine how many regex matches indicate that a PICO element is part of an experiment described within a publication, rather than a match to a reference to other research.

These caveats suggest that while automated approaches are useful, they cannot completely replace human reviewers; a SOLES project will still require some human input to ensure quality of the data that is output.

## Future development

Our key short-term goal for SOLES is to continually optimise the automated approaches we use, improving the performance of existing tools and optimising our approaches to take advantage of new tools which may become available. In particular, we recognise the growing need for high-level database management functionality for running automated scripts and updating records, and the implications this has in maintaining the quality of synthesised data.

In the long-term, we hope to focus on the interface between SOLES and SRs derived from SOLES projects. Interoperability between SOLES data visualisation apps and SR platforms such as the Systematic Review Facility (SyRF; [62]) strengthens the community and crowd approach to working collaboratively. This would benefit both SOLES developers and researchers conducting SRs, as additional data collected from derived SRs could feed back into a broader SOLES overview. For SRs derived from SOLES that share similar methodology, further efficiency gains could be made by developing an overarching master protocol with shared workflows and infrastructure for in-depth annotation, data extraction, analysis, reporting and visualisation. The SOLES approach and its flexibility means it is ideal to display outputs from other types of evidence synthesis, for example, network meta-analysis, systematic maps and scoping reviews.

Having developed SOLES across several research domains, we hope to expand our approach and triangulate diverse evidence streams including a greater focus on clinical evidence and results from *in vitro* experiments. Furthermore, the same interventions, drug targets and underlying biological pathways may be studied within different contexts. Combining evidence from across SOLES projects could enable us to uncover hidden links across typically distinct research silos. While aspects of SOLES overlap with evidence gap maps, we aim to leverage novel automation tools to support and expand our approach going forward to provide more advanced insights and drive hypothesis generation.

Finally, we seek greater collaboration with key research stakeholders who may benefit from the data that a SOLES can provide, such as laboratory researchers, clinicians and other healthcare decision makers. Only with effective collaboration can we ensure that SOLES are fit for purpose and beneficial to the wider scientific community.

## Concluding remarks

Systematic Online Living Evidence Summaries are an emerging approach to benefit multiple key stakeholders in the evidence synthesis process, including primary researchers, evidence synthesists, information specialists and statisticians, funders, regulatory agencies and policymakers.

We believe this approach can bring these stakeholders together to work collaboratively to answer pressing research questions in biomedicine, forming communities to bridge the translational gap and make the most effective use of research.

The approach is flexible, data processing pipelines can be expanded to add new validated tools as they become available, to meet the diverse and growing needs of evidence stakeholders in biomedicine.

## Data Availability

We did not generate nor analyse any new data for this manuscript.

## Abbreviations

MND: Motor Neuron Disease
PICO: Population, Intervention, Comparison and Outcomes
SOLES: Systematic Online Living Evidence Summaries
